# Omega-3 Fatty Acids and FFAR4

**DOI:** 10.3389/fendo.2014.00115

**Published:** 2014-07-16

**Authors:** Da Young Oh, Evelyn Walenta

**Affiliations:** ^1^Division of Endocrinology and Metabolism, Department of Medicine, University of California San Diego, La Jolla, CA, USA

**Keywords:** omega-3 fatty acids, FFAR4, anti-inflammation, insulin resistance, obesity

## Abstract

The beneficial roles of omega-3 fatty acids (ω3-FAs) on obesity, type 2 diabetes, and other metabolic diseases are well known. Most of these effects can be explained by their anti-inflammatory effects triggered through their receptor, free fatty acid receptor 4 (FFAR4) activation. Although the whole mechanism of action is not fully described yet, it has been shown that stimulation of ω3-FA to FFAR4 is followed by receptor phosphorylation. This makes FFAR4 to be capable of interacting with β-arrestin-2, which in turn, results in association of β-arrestin-2 with TAB1. This stealing of an important partaker of the inflammatory cascade leads to interruption of the pathway, resulting in reduced inflammation. Besides this regulation of the anti-inflammatory response, FFAR4 signaling also has been shown to regulate glucose homeostasis, adiposity, gastrointestinal peptide secretion, and taste preference. In this review, we summarize the current knowledge about the interaction of ω3-FAs with FFAR4 and the consequent opportunities for the application of ω3-FAs and possible FFAR4 targets.

## Introduction

Free fatty acids (FFAs) serve both as a source of energy and as signaling molecules that regulate energy homeostasis and other physiological processes (1, 2). Previous studies proposed that lipotoxic stress from long-chain saturated FFAs is a major cause of JNK activation and therefore insulin resistance in obesity (3, 4). It has also been reported that long-chain saturated FFAs, but not polyunsaturated FFAs, induce inflammatory responses in macrophages (5). Among polyunsaturated FFAs, omega-3 fatty acids (ω3-FAs) have been recognized for their beneficial effects on human health (6). These beneficial effects were found in inflammatory diseases, cardiovascular diseases, and hepatic lipid metabolism, as well as glucose homeostasis and insulin sensitivity (7–10). However, the detailed mechanisms underlying the beneficial effects of ω3-FAs have not been completely defined to date.

It is known that the ω3-FAs, such as α-linolenic acid (α-LA), docosahexaenoic acid (DHA), and eicosapentaeonic acid (EPA) are endogenous ligands for the free fatty acid receptor 4 (FFAR4) (11–13). FFAR4, also known as G protein-coupled receptor 120 (GPR120), is a seven transmembrane receptor and was first reported as an orphan GPCR in 2003 (14). FFAR4 exists as two splice variants in humans (15, 16), but only the shorter variant has been found in rodents and cynomolgus monkeys (17). Consistent with the pleiotropic effects of ω3-FAs, FFAR4 has been implicated in diverse processes including anti-inflammation, insulin sensitization, release of gut peptides, and alteration of food preference (12, 13, 18–21). The wide range of processes that can be positively influenced by FFAR4 makes this receptor of potential importance in the prevention and treatment of metabolic diseases.

## Mechanisms

**Figure 1:**
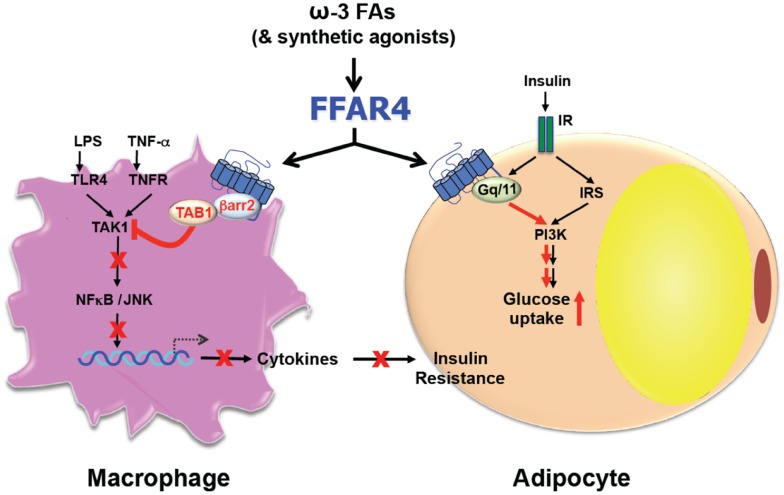
**Free fatty acid receptor 4 functions as an ω3-FA receptor/sensor to mediate broad anti-inflammatory and insulin sensitization effects**.

Consistent with roles in diverse processes, FFAR4 is expressed ubiquitously including lungs, colon, small intestine, brain, thymus, adipose tissue, taste buds, skeletal muscle, heart, and liver (12, 18, 21, 22). However, the expression pattern differs between species and also depends on the method of detection (23). Furthermore, the expression in skeletal muscle, heart, and adipose tissue is upregulated by a high fat diet (HFD) (18, 19, 22). Despite the variation of expression, FFAR4 seems to transduce ω3-FA signaling always through one of two pathways that involve either G_αq_ or β-arrestin-2 (12, 13) (Figure 1). FFAR4 coupling to G_αq_ induces a rise in intracellular Ca^2+^ (12, 21) without affecting the level of cyclic AMP (12). Alternatively, FFAR4 can respond to ω3-FAs by recruiting cytosolic β-arrestin-2 to the plasma membrane, leading to internalization of the FFAR4/β-arrestin-2 complex (13). After internalization, β-arrestin-2 then directly associates with TGF-β activated kinase 1 (TAK1) binding protein (TAB1), which is an adaptor molecule for the pro-inflammatory kinase TAK1 (24). In this way, β-arrestin-2 sequesters TAB1 from TAK1, leading to inactivation of TAK1 and abrogation of signaling to the inflammatory key-players IKKβ/NFκB and MKK4/JNK/AP1 (13). Besides blocking the inflammatory pathway via the TAB1/TAK1 complex, FFAR4 activation by DHA also stimulates cytosolic phospholipase A_2_ (cPLA_2_) and prostaglandin-endoperoxide synthase 2, also known as COX-2 (25). This leads to increased production of prostaglandin E_2_ (PGE_2_), which in turn, inhibits NFκB signaling through the prostaglandin E receptor 4 and therefore reduces inflammation in macrophages as well (25).

After activation by ligands, GPCRs are phosphorylated to provide binding opportunities for certain G proteins and arrestins (26, 27). Investigating the human short isoform of FFAR4, Sánchez-Reyes et al. (28) found that DHA and α-LA induce phosphorylation of FFAR4 by protein kinase C (PKC), leading to increase of intracellular Ca^2+^ concentration. However, recent studies of the long isoform of FFAR4 by Burns et al. (29) showed that basal as well as heterologous FFAR4 phosphorylation is mediated by PKC, while DHA-induced phosphorylation is accomplished by GPCR kinase 6 (GRK6). Interestingly, mutation of the FFAR4 C-terminal phosphorylation sites leads to enhanced G_αq/11_ signaling while impairing β-arrestin-2 recruitment to the cell membrane. However, Burns et al. (15) also found that the human short and long isoforms differ in their basal levels of phosphorylation, raising the possibility that the short isoform is more constitutively active. In the activated state though, both isoforms show comparable extent of phosphorylation. While the long isoform is proposed by Watson et al. to bind with greater affinity to β-arrestin-2 (16), a partial loss of function is proposed for the long isoform by Hirasawa et al. (12) and Moore et al. (17).

## Tissue-Specific Functions

Due to the divergence of the reported functions, FFAR4 shows tissue-specific activities, which needs to be taken into account particularly for the development of pharmaceutical intervention. Therefore, a tissue-specific knockout (KO) mouse would be of high interest to discover precisely where certain effects of FFAR4 impact. This is critical for the development of agents that target inflammation, insulin resistance, and as a result, type 2 diabetes. However, no conditional FFAR4 KO mouse is available to date and mouse models cannot be used to investigate the function of the long isoform of FFAR4, which is unique to humans. Given the known differential phosphorylation of the two isoforms (15), their functions and regulation likely differ, which will have to be addressed in human tissues and cell lines.

### Adipogenesis

Free fatty acid receptor 4 expression is undetectable in preadipocytes (18, 30) but increases during adipogenic differentiation (18, 30), and becomes highly abundant in mature adipocytes and adipose tissue (13, 18, 19). The expression in adipose tissue is further increased by diet-induced obesity in mice and humans (18, 19). Conversely, knockdown of FFAR4 using siRNA reduces the expression of adipogenic markers and therefore impairs the accumulation of lipids in 3T3-L1 adipocytes (18). Although these findings suggest a pro-adipogenic function of FFAR4, FFAR4-deficient mice are actually more prone to diet-induced obesity than wild type littermates (19), consistent with an anti-obesity function of FFAR4.

It should be mentioned that discordant phenotypes of FFAR4 KO mice have been reported using two different mouse models. Thus, the body weight of FFAR4 KO mice is either increased (19) or unaffected (31) on HFD, and the insulin sensitivity is either decreased (31) or unaffected (19) on chow diet between wild type and KO mice (13, 32). Being all on the same C57BL/6 background, the mice seem to be either the N or the sub-lines, which might explain the varying findings. However, all models consistently establish the key site of FFAR4 action to be the adipose tissue, where ω3-FAs clearly exert FFAR4-dependent anti-diabetic effects in adipocytes and macrophages (13, 18, 19).

### Inflammation

Omega-3 FAs have tissue-specific as well as systemic anti-inflammatory effects (33). FFAR4 is key to these benefits in adipose tissue macrophages, which abundantly express this receptor. Furthermore, FFAR4 expression in macrophages is induced upon obesity. It was shown that its activation by ω3-FAs in mice fed with HFD supplemented with ω3-FAs leads to the suppression of macrophage infiltration into adipose tissue (13). Additionally, ω3-FAs shift the distribution of macrophages in favor of the anti-inflammatory M2 macrophages at the expense of the pro-inflammatory M1 macrophages (13). In the brain, intracerebroventricularly injection of either ω3- or ω9-FAs induces FFAR4/β-arrestin-2 coupling followed by the release of TAK1 from TAB1, leading to attenuation of the inflammatory pathway (34). Additionally, Wellhauser et al. showed the anti-inflammatory effects of FFAR4 activation in immortalized hypothalamic neurons (35). This is of importance as on HFD that hypothalamus becomes inflamed and fails to regulate energy homeostasis through regulating glucose handling, feeding, and therefore, body weight.

Finally, FFAR4 activation also leads to improvement of non-alcoholic fatty liver disease (NAFLD) in children (36). In more detail, Nobili et al. detected FFAR4 expression in hepatocytes, liver macrophages, and liver progenitor cells. DHA treatment of children suffering from NAFLD increased the FFAR4 expression in hepatocytes and reduced nuclear NFκB translocation in hepatocytes and liver macrophages, as well as reduced hepatic progenitor cell activation and the number of inflamed macrophages in the liver (36). Accordingly, ω3-FAs also protect liver from ischemic reperfusion injury (IRI), a complication of liver surgery. Treatment with Omegaven^®^, a pharmaceutical ω3 formulation, reduces NFκB and JNK response and shifts the macrophage population from M1 to M2 (37).

### Insulin signaling

Besides the food intake and digestive influences of FFAR4 activation by ω3-FAs, increased gut glucagon-like peptide 1 (GLP-1) secretion increases pancreatic insulin secretion, leading to enhanced glucose uptake in skeletal and cardiac muscle (38, 39). Whether FFAR4/ω3-FAs play a cell-autonomous role in muscle, the major site of insulin-stimulated glucose uptake and systemic insulin action (40, 41), has yet to be investigated in detail, as Cornall et al. (22) found FFAR4 expression increased in skeletal and cardiac muscle of rats on HFD, while no expression was detected in L6 myocytes (13). Nevertheless, it has been shown that FFAR4 activation by ω3-FAs leads to insulin sensitization *in vivo* and also alleviates glucose intolerance in diet-induced obese mice (13, 19). Although the anti-inflammatory effects of ω3-FAs require FFAR4 coupling to β-arrestin-2, the hypoglycemic effect of insulin requires G_αq_ signaling that leads to translocation of the glucose transporter GLUT4 (13). Conversely, FFAR4 KO mice show reduced phosphorylation of IRβ and IRS-1 in white adipose tissue and of IRS-1 and -2 in liver, which are all important regulators for glucose uptake (19). Not surprisingly, these mice develop hyperglycemia, glucose intolerance, and insulin resistance when challenged with a HFD (19).

### Gastrointestinal regulation

Gastrointestinal peptides are known to regulate food intake, energy metabolism, and body weight (42–47). Activation of FFAR4 by ω3-FAs has been shown to either reduce or induce the secretion of several gastrointestinal peptides. For example, FFAR4 activation decreases the secretion of ghrelin, an endogenous growth hormone secretagogue that stimulates hunger (48, 49). Additionally, FFAR4 was shown to induce the secretion of GLP-1 and cholecystokinin (CCK) (12, 20, 21). GLP-1 is an insulinotropic, anorectic peptide that reduces gastric emptying and motility (43, 44). CCK is similar to GLP-1 in that it inhibits gastric motility, but in addition it inhibits gastric secretion while promoting pancreatic secretion and gallbladder contraction (50). It was shown by Stone et al. that FFAR4 activation leads to decreased somatostatin secretion (51), which in turn, can increase insulin and glucagon secretion as well as gastric emptying. This is in sharp contrast to the recent finding by Suckow et al. of increased glucagon secretion in FFAR4 KO mice (32).

Unfortunately, some of these findings are contradictory. Although several groups demonstrated FFAR4 expression in islets (52–54), there is no consensus on the site of action, like influencing secretion of GLP-1, glucagon, or somatostatin, and even the cell type in which it is expressed most, is contradictory (21, 32, 51).

### Taste preferences

Free fatty acid receptor 4 is abundantly expressed in several types of taste bud cells (55–57) and therefore can be activated directly by dietary ω3-FAs. Although detailed investigation is needed, it seems that FFAR4 might dictate spontaneous preference for specific dietary fats (31) that are abundant in energy-dense foods (58). However, Ozdener et al. (57) found that CD36 is the primary receptor for fat taste, while FFAR4 senses excess supply of FAs as in HFD.

## Synthetic Ligands of FFAR4

Like FFAR4, FFAR1 (a.k.a. GPR40) is a receptor for long-chain ω3-FAs. Although the two receptors share endogenous as well as several synthetic agonists (11), like the PPARγ derivate GW9508 (59, 60), a few ligands are known to be partly or more selective for FFAR4 than FFAR1, namely grifolic acid, NCG21, GSK137647A, and TUG-891 (61–64). However, the relatively low efficacy of these known synthetic agonists in several measured outputs raises questions whether these can be therapeutically relevant molecules.

## Perspective

Solving the mechanism of FFAR4/ω3-FAs might lead to a more directed and therefore more potent way of fish oil supplementation. But besides that, the understanding of the mechanism hopefully will lead to the development of a more FFAR4-specific, high affinity agonist. The direct activation of FFAR4 itself is of special interest, as FFAR4 is a “druggable” GPCR. Unfortunately, to date, there are no FFAR4-specific agonists that spare other GPCRs like FFAR1, which leads to off-target effects. It might be possible to develop an FFAR4 agonist that is tissue-, pathway-, or isoform-specific and therefore provides anti-inflammatory/insulin-sensitizing effects. Taken together, finding of an FFAR4-specific agonist will be a new therapeutic approach for the treatment of both metabolic and inflammatory diseases.

## Conflict of Interest Statement

The authors declare that the research was conducted in the absence of any commercial or financial relationships that could be construed as a potential conflict of interest.
